# Essay on the Tetanus Rabiensis; or, Enquiry into the Causes of the Accidents Which Sometimes Ensue from the Bites of Animals, Said to Be Rabid; with Some Hints Respecting the Best Method of Preventing or Curing These Affections

**Published:** 1810-01

**Authors:** M. G. Girard

**Affiliations:** Lyons


[The following report of a work, with the aannexed title, has been presented to the French Institute. It is drawn up by Etienne Sainte Marie, M. D.]

*Essay on the Tetanus Rabiensis; or, Enquiry into the Causes of the Accidents which sometimes ensue from the Bites of Animals, said to be Rabid; with some Hints respecting the best Method of preventing or curing these Affections.* By M. G. GIRARD, M. D. of Lyons.

THE author of the work in question endeavours to prove, that the morbid affection commonly called rabies, or hydrophobia, is imperfectly designated by these terms; that hydrophobia is not a disease in itself, but merely a symptom of disease; that this opinion was probably entertained by Hippocrates, who does not in any of his works speak of hydrophobia, although he was perfectly well acquainted, as various passages amply prove, with all the symptoms attributed to the disease so called at present; that the saliva of a rabid animal, when deposited by a bite on the body of another animal, is not the cause of the the affections which the latter experiences some time afterwards; that these affections rather belong to the bite itself, which, by disorganising the skin and the subjacent parts, disturbs the circulation of the fluids, causes congestions, and determines irritation in the nerves, spasms, &c.: finally, that rabies, called the hydrophobia, communicated by infection, is frequently nothing else than a traumatic maxillary tetanus; or, to speak a language intelligible to all the world, a spasm of the jaws, produced as the consequence of a wound.

Every fact which can possibly favour these opinions, is cited in the course of the work; the materials being partly collected from the best medical works, and partly furnished by the author from his own practice. Two celebrated physicians of the present day, Dr. Percival, of London [Manchester], and Dr. Rush, of Philadelphia, have declared hydrophobia to be a tetanus. These gentlemen published their opinions nearly at the same period, without being acquainted with the sentiments of each other. With respect to M. Girard, his ideas on the subject were ready for the press, when chance brought in his way a periodical work, in which a mere notice was given of the opinions of Drs. Percival and Rush.

We frequently observe that men, who have been bitten by rabid animals, do not contract hydrophobia; while others are seized with it, when bitten by animals in a healthy state. There is no one who cannot adduce facts in support of the former of these hypotheses. If those in support of the latter are fewer in number, we find several in medical works; and M. Pinel, in his Nosographie Philosophique, has given a very strong one. A man, who was a prey to domestic misfortunes, was bitten by his horse. He was seized with hydrophobia; but the horse, which was carefully watched, did not become rabid. M. Girard cites, in the first place, similar examples, in order to prove that there exists in the disease, called hydrophobia, something which we have not as yet been able to discover. He afterwards enumerates the symptoms peculiar to hydrophobia and to tetanus; demonstrates their analogy, and concludes that the two diseases are completely identical. In both cases, in fact, we observe between the period of the bite and the first developement of the symptoms, a longer or shorter interval; a dull pain in the flesh, and sometimes a swelling in the cicatrix, on the approach of the crisis; a spasmodic irradiation, extending from the wounded part to the neck; spasms in the jaws jaws and pharynx; and difficulty, or even absolute incapacity, to swallow: a repugnance to drink, carried, in some instances, to horror; a copious flow of thick saliva; a grinding of the teeth; general or partial convulsions, coming on in paroxysms, agitation, &c.

I shall not follow the author into his train of argument in support of his theory, but content myself with quoting such of his experiments as seem to be the most conclusive and ingenious.

He attempted to inoculate the rabies on a dog, with a lancet impregnated with saliva, furnished by a girl labouring under hydrophobia: the animal received several punctures in the thigh, and yet continued to enjoy good health. Similar experiments, made by M. Huzard, professor in the veterinary school at Alfort, and M. Gibaud, surgeon to the Hotel Dieu of Paris, gave similar results.

M. Gerard next wounded dogs with the bites of cats and other animals; he made in different parts of their bodies deep and irregular wounds, with variously shaped and pointed instruments; and he succeeded in producing grinding of the teeth, difficulty in swallowing, repugnance to food and drink, suffocation, an abundant flow of frothy saliva, i.e. most of the symptoms which are common to rabies and to tetanus.

Although the author establishes his opinion in a manner equally solid and ingenious, his proofs are still by no means satisfactory. It is very true, that rabies and tetanus resemble each other much. I will even go farther and say, that there may be a traumatic rabies; or, to preserve the technical and accurate expressions of the author, a *tetanus rabiensis;* such as is caused by the bites of animals merely in a furious or irritated state. But he must go farther, if he wishes to have a precise notion of true or essential rabies. This dreadful disease is a deprivation of sensibility: it consists in fits of furious mania, with or without delirium; the intervals being spent in profound melancholy. Now we find nothing similar in tetanus. Delirium is rare in this affection; and when it does occur, it is a confusion in the ideas, like what is observed in fevers. It is not, as in rabies, an error almost entirely confined to the taste, appetite, affections, or sentiments; in a word, to that order of movements, which are regulated by sensibility. In my opinion, rabies will be one day classed, in imitation of the ancient method, among cases of insanity; and it will be regarded as a variety of sentimental melancholic mania. M. Pinel, who, in the first edition edition of his Nosographie, had placed rabies among spasmodic diseases, has already, in his subsequent editions, restored it to the vesaniæ. The day will, perhaps, soon arrive, when a more perfect nosology will not, separately, classify rabies, mania without delirium, melancholia, low nervous fever, hypochondriasis, hysteria, nostalgia, spleen, or the English disease, &c.—all of them affections, in which it is likely that we shall discover the spinal marrow, the grand intercostal nerve, the solar plexus, and all the organs which obey these central points of sensibility, to be more or less implicated.

M. Girard concludes his work with some very judicious observations on the treatment of tetanus rabiensis: even in the use of the methods indicated as efficacious against rabies, he finds a support to his opinion, as to the nature of the disease. He proves, that if the treatment called local, be more advantageous than any other, it is because, by means of caustics, scarifications, and the methods used to produce an abundant suppuration, we produce a simple, instead of a deep, tortuous, and irregular wound.

The work deserves to be attentively read and considered; it is not one of the empty compilations of an hour; it is a succinct, original, and ingenious treatise on rabies; and, from the novelty of the views it presents, must excite the interest of every medical practitioner. It is composed in that philosophic spirit, which, although long displayed with success in other branches of science, has hitherto, from a deplorable fatality, shed but little of its influence on medical science.

The most important service we can render to the healing art, is to simplify the study of medicine, and to generalise more and more those actions, which may evince the laws of diseases.

M. Girard has attempted this, as far as rabies is concerned; he has cleared the history of this affection from all the absurd stories, which the love of the marvellous, popular belief, the credulity of medical men, or hasty conclusions, had introduced. He subtracts one from the number of our present diseases; but, in the order of spasmodic affections, he has given us an additional species. I fear, lest this system, specious as it is, should mutilate rather than simplify truth; and this doubt I have already ventured to suggest.

If it be clearly ascertained that the saliva of rabid animals cannot communicate rabies, which is perhaps the case, we shall approach hydrophobic patients with more conconfidence. We can bestow on them more assiduous, intelligent, and affectionate attendance; we shall have no occasion to fear the effects of their bites; but it is rare that a human being, in this unhappy state, attempts to bite. It cannot be asserted, however, as has lately been said, that patients of this description never bite at all. I was witness to a contrary instance.

Upon the whole, I have no doubt that, in addition to the approbation bestowed on M. Girard's labours by the Institute, he will acquire the esteem and gratitude of every friend to humanity.

*Moniteur, Nov.* 21, 1809.

